# The Relation of Over-Nutrition after the Acute Fevers of Childhood to Bone Disease

**Published:** 1885-01

**Authors:** 


### The Relation of Over-Nutrition after the Acute Fevers of Childhood to Bone Disease.

Dr. Jacobi said he would refer to a class of cases which were not very uncommon, and which were interesting because of their connection with a number of physiological and pathological questions. A very simple and illustrative case was the following:

Some time ago a girl, eleven or twelve years of age, was present at his clinic for a swollen right humerus at its lower portion. The swelling was very slightly painful. There was a cicatrix about the middle of the arm, which had formed about six months before, after a sinus had lasted six years. A fistula opened an inch and a half above the elbow, on the anterior aspect, which led down to about the middle portion of the epiphysis apparently extending to the periosteum only. It was stated that the humerus began to swell when the child was four years old, and very soon after she had gone through a very severe attack of typhoid fever. The question arose, Had this swelling of the bone and periosteum anything to do with the typhoid fever? Dr. Jacobi thought it had, for reasons which he would state. While he might not be able to say anything that was not known to every person present, still the case was of interest in connection with a number of others which he had seen, and which were very interesting to him, particularly so because they opened up the question of quite a large number of cases of a similar description. There was one peculiar fact in the development and growth of children, which was known to physicians and also to the laity, that children not only appeared very tall after having gone through a severe illness, and particularly through a severe infectious disease, but they were really taller than before the sickness, and they grew very rapidly for a short time during and after such infectious disease. The growth or tallness was not only apparent, from the patient having become thin, but by measurement it could be shown that they actually were taller. The body became taller by an elongation of the bones; the bones grew by a rapid proliferation about the cartilage which separated the epiphysis from the shaft. If the bone grew, it must be in consequence of a nutritive process, which might become an irritative process, in that neighborhood. And the question arose whether high fevers, and infectious fevers particularly, had not the effect of producing such irrita-

18

tive disorder as proved, under certain circumstances, a cause of increased nutrition. Observation showed that after all cases of infectious disease, in particular the epiphyses and the adjoining cartilages were very hyperæmic. In such localities, if a post mortem examination were made, the blood would be found to ooze out, and where there was much blood there was at least an opportunity for over-nutrition. In rhachitical bones we knew that the intense growth and thickness were due to such over-nutrition. Thus it was that after most infectious fevers not only the epiphyses were apt to grow thicker, but also the diaphyses to grow longer, in consequence of the nutritive irritation of the cartilage (and periosteum). In cases in which the nutritive disorder, the hyperæmia, was not limited to its physiological condition, where it was a little more than physiological, it became pathological. In most cases the over-nutrition and growth ceased after awhile and returned to the normal state, but in others they were carried to such an extent as to become pathological and cause necrosis. Such over-nutrition of the epiphyses was one of the forms of so-called "growing-pain." Growing-pains occurred very frequently after a severe illness, and especially after a severe attack of an infectious fever, and were due to hyperæmia which might amount to inflammation. The other forms of "growing-pain" were either rheumatic or neuralgic in character.—*The Obstetric Gazette.*

